# Phenotypic Features and Genetic Findings in a Cohort of Italian *Pseudoxanthoma Elasticum* Patients and Update of the Ophthalmologic Evaluation Score

**DOI:** 10.3390/jcm10122710

**Published:** 2021-06-19

**Authors:** Federica Boraldi, Vittoria Murro, Francesco Demetrio Lofaro, Dario Pasquale Mucciolo, Sonia Costa, Laura Pavese, Daniela Quaglino

**Affiliations:** 1 Department of Life Science, University of Modena and Reggio Emilia, Via Campi 287, 41125 Modena, Italy; federica.boraldi@unimore.it (F.B.); francescodemetrio.lofaro@unimore.it (F.D.L.); sonia.costa@unimore.it (S.C.); 2 Department of Neuroscience, Psychology, Drug Research and Child Health, University of Florence, Eye Clinic, Viale Pieraccini 6, 50139 Florence, Italy; vittoria.murro@unifi.it (V.M.); dario.mucciolo@gmail.com (D.P.M.); laurapavese4@gmail.com (L.P.)

**Keywords:** PXE, phenotype, Phenodex, atrophy, comet lesion, pattern dystrophy, *ABCC6*

## Abstract

Background: *Pseudoxanthoma elasticum* (PXE) is a rare ectopic calcification genetic disease mainly caused by *ABCC6* rare sequence variants. The clinical phenotype is characterized by typical dermatological, ophthalmological and cardiovascular manifestations, whose frequency and severity are differently reported in the literature. Methods: A retrospective study was performed on 377 PXE patients of Italian origin, clinically evaluated according to the Phenodex Index, who underwent *ABCC6* biomolecular analyses. Moreover, 53 PXE patients were further characterized by in-depth ophthalmological examinations. Results: A total of 117 different *ABCC6* rare sequence variants were detected as being spread through the whole gene. The severity of the clinical phenotype was dependent on age, but it was not influenced by gender or by the type of sequence variants. In-depth ophthalmological examinations focused on the incidences of *coquille d’oeuf*, comet lesions, pattern dystrophy-like lesions, optic disk drusen and posterior-pole atrophy. *Conclusion*: Given the large number of patients analyzed, we were able to better evaluate the occurrence of less frequent alterations (e.g., stroke, myocardial infarction, nephrolithiasis). A more detailed description of ophthalmological abnormalities allowed us to stratify patients and better evaluate disease progression, thus suggesting a further update of the PXE score system.

## 1. Introduction

*Pseudoxanthoma elasticum* (PXE, OMIM 264800) is a rare genetic disease (1:25,000–1:50,000) characterized by progressive calcification of the elastic component within soft connective tissues [1]. It mainly affects the skin, blood vessels and the eyes, with the latter being the most severely affected in terms of the impact on a patients’ quality of life. Microcalcifications can be also detected in several other organs [2], which do not appear to be clinically affected by the disease and exhibit laboratory parameters within a normal range.

Skin alterations are usually in the form of round yellowish papules that, over time, form large plaques frequently associated with areas where skin becomes wrinkled and redundant [3]. 

Cardiovascular manifestations (e.g., diminished or absent peripheral vascular pulsations, accelerated atherosclerosis, premature *intermittent claudication* and possibly also hypertension) can already be observed before the third or fourth decade of life, and are mainly due to the calcification of the elastic laminae of medium sized arteries [4]. A great difference in the incidence of myocardial and cerebral infarction, or stroke, has been reported in a number of studies [5,6]. Moreover, due to the frailty of calcified submucosal vessels, about 10% of PXE patients may exhibit gastrointestinal hemorrhages [7]. 

In PXE, calcification of the Bruch’s membrane leads to the typical ocular alterations, usually described as *peau d’orange*, that generally precede angioid streaks [8,9]. Over the course of the disease, almost all PXE patients develop angioid streaks and choroidal neovascularization (CNV), which are responsible for progressive decreased central visual acuity and can lead to legal blindness [10,11]. 

Although PXE has been associated [12] with mutations in the *ABCC6* gene since 2000, the clinical expression of the disease is highly heterogeneous, with considerable variation in age of onset, progression and severity, even within the same family and in the presence of identical DNA sequence variants [13,14,15]. At present, 539 different variants have been described in the *ABCC6* gene (https://databases.lovd.nl/shared/genes/ABCC6 (accessed on 25 March 2021)), of which more than 350 are rare and considered pathogenic [16]. 

The aim of our study was to retrospectively investigate a large cohort of PXE patients, all of Italian origin, to better evaluate their phenotype, while also looking at less frequent alterations (e.g., stroke, myocardial infarction, nephrolithiasis). Moreover, given the impact that visual impairment/loss has on PXE patients and the improvement in retinal imaging techniques, we performed a more detailed description of ophthalmological abnormalities on a subgroup of patients so as to better evaluate disease progression and improve PXE patients’ counselling.

## 2. Materials and Methods

### 2.1. PXE Patients and Genetic Analyses

In the present study, a cohort of 377 PXE patients of Italian origin was retrospectively investigated (from 2008 to 2018), and data were collected at the time of biomolecular diagnoses. Clinical diagnosis was performed on the observation of typical cutaneous and/or ocular manifestations. Moreover, to confirm clinical diagnosis, pathogenic sequence variants were investigated by Sanger sequencing of the 31 exons of the *ABCC6* gene on genomic DNA isolated from whole blood in EDTA (QIAamp blood kit, Qiagen GmbH, Hilden, Germany), according to standard procedures [17]. Long-range PCRs using primers positioned at the flanking regions were conducted to detect shorter PCR fragments compared to controls, allowing us to identify large deletions [17]. Patients with unidentified variant alleles also underwent multiplex ligation-dependent probe amplification (MLPA) analysis, as already described [18].

The severity of the clinical phenotype (i.e., skin, eye, cardiac, gastrointestinal, renal and vascular manifestations) was globally evaluated according to the Phenodex index, which was updated by Legrand and coworkers [19]. Patients who were initially scored according to the first published Phenodex index [20] were later reconsidered in light of new parameters [19].

### 2.2. In Silico Analysis for Pathogenisity Predictions of Rare Sequence Variants

Rare sequence variants were named according to the nomenclature recommendations of the Human Genome Variation Society (HGVS) (http://www.hgvs.org (accessed on 15 January 2021)).

All rare sequence variants found through genetic testing were classified into five classes, according to the guidelines of American College of Medical Genetics and Genomics and the Association for Molecular Pathology (ACMG/AMP) [21]: benign (C1); likely benign (C2); uncertain significance [(VUS) (C3)]; likely pathogenic (C4); and pathogenic (C5). In particular, we used the VarSome platform (https://varsome.com/ version: 9.1.2 (accessed on 15 January 2021)), a human genomic variant search engine, which includes information from several external databases (e.g., disease databases, scientific and medical literature, in silico prediction programmes, functional data) and evaluates the submitted variant according to the five classes [22]. Moreover, for new missense variants, we performed additional in silico analyses with software not included in the free version of VarSome: Polyphen-2 (Polymorphism Phenotyping v2) to assess the impact of variants on protein structure or function [23], where a value close to 1 indicated a high “security” of the prediction. Panther was used to predict the effect of non-synonymous substitutions while looking at sequence conservation using multiple sequence alignments [24], and Align GVGD was used to combine the biophysical characteristics of amino acids and protein multiple sequence alignments [25]. The GVGD score goes from 0 (no pathogenic) to 65 (highly predicted as pathogenic).

### 2.3. Ophthalmologic Examinations

All 377 PXE patients, spread throughout Italy, underwent routine ophthalmological examination, OCT or fluorescein examination. 

Moreover, among patients who visit the Eye Clinic in Florence in the period from 2012 to 2016, 53 PXE patients, the clinical diagnosis of whom was confirmed by two pathogenic ABCC6 rare sequence variants, underwent in-depth ophthalmological examination with advanced fundus imaging techniques: wide-field color fundus photographs, wide-field fundus autofluorescence examination (FAF) and OCT-angiography (OCT-A). 

Atrophy was defined as dropout of the retinal pigment epithelium (RPE) and visibility of the choroidal vessels on fundus color photographs, clearly demarcated hypo-autofluorescent areas on FAF. On OCT, the loss of the ellipsoid zone and RPE band, and associated hyper-transmission posterior to Bruch’s membrane, were considered to support the diagnosis of atrophy. We classified the extensive atrophic area which involved the entire posterior pole as posterior pole atrophy, including atrophy surrounding the CNV when the extension affected the entire posterior pole.

Active or inactive CNV was defined by the following findings: intra- or sub-retinal fluid on OCT imaging (in selected cases, in combination with leakage on angiography), intra- or sub-retinal hemorrhage, signs of sub-retinal fibrosis on fundus color and/or OCT images and the presence of CNV signs using OCT-A.

Patients were diagnosed with pattern dystrophy-like changes (PD) by at least two of the authors (VM, LP and DPM), based on clinical, angiographic, fundus autofluorescence and OCT findings and, when possible, these patients were divided into PD subgroups according to Gass pattern dystrophy classification [26]. 

### 2.4. Statistical Analyses

Data were analyzed with GraphPad Prism software, version 8 for MAC (GraphPad Software, San Diego, CA, USA). Correlation between age and Phenodex score was assessed by linear regression analysis, with 95% confidence intervals. The incidence of clinical characteristics between PXE patients and the general population was assessed using the Pearson chi-square test and the Fisher’s exact test when the number of cases in a subgroup analysis was too low. *p* < 0.05 was considered statistically significant. 

## 3. Results and Discussion

### 3.1. ABCC6 Rare Sequence Variants 

**Figure 1:**
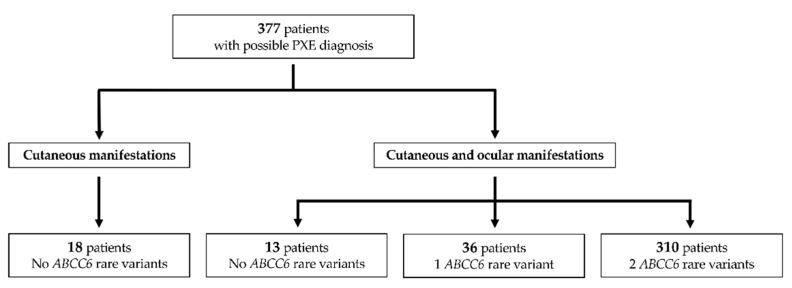
Study flowchart of patients investigated in the present study.

Biomolecular analyses of the *ABCC6* gene were performed on a cohort of 377 who were exhibiting clinical manifestations supporting the possible diagnosis of PXE (Figure 1).

In particular, 18 patients (mean age 52 ± 19 yrs) exhibited skin alterations suggestive of PXE and were negative for *ABCC6* rare sequence variants. Skin biopsies were available for 6/18 patients, and ultrastructural examination revealed the absence of the typically mineralized elastic fibers, suggesting that clinically observed skin lesions were likely due to the age of subjects or other diseases with a dermatological overlapping phenotype (e.g., *Cutis laxa*, Ehlers Danlos, Buschke Ollendorff). 

The remaining 359 patients were clinically diagnosed as having PXE based on two major criteria (i.e., cutaneous and ocular manifestations). Biomolecular analyses of the *ABCC6* gene failed to detect rare sequence variants in 13 patients, whereas one and two rare sequence variants were found in 36 and 310 patients (98 patients homozygous and 212 compound heterozygous, as confirmed by segregation analysis), respectively (Figure 1).

The absence or presence of one *ABCC6* rare sequence variant in patients with skin and ocular manifestations indicates that other genes or undetected *ABCC6* deep intronic variants cannot be excluded. As it is a retrospective investigation, a limit of the present study is that biomolecular analyses were limited to the *ABCC6* gene. 

In 310 patients, 117 different rare sequence variants were detected, and they were distributed on the whole *ABCC6* gne (Table 1 and Table S1). In total, 96/117 (82%) had been already published, while the remaining 21 (18%) were reported in this study (Table 1 and Table S1).

**Figure 2:**
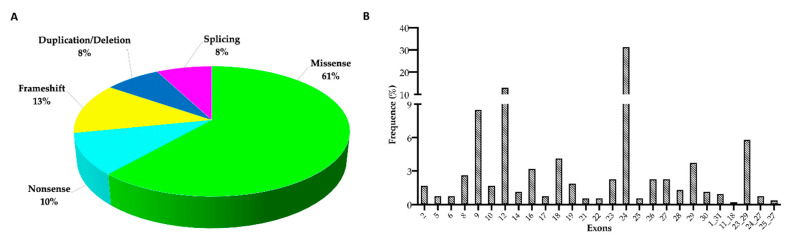
Type and frequency of *ABCC6* rare sequence variants. (**A**) Distribution of the different types of rare sequence variants found in the *ABCC6* gene. (**B**) Frequency distribution of *ABCC6* rare sequence variants at exon-level in Italian PXE patients.

Figure 2A,B shows the frequency of the different categories of rare sequence variants and their distribution along the *ABCC6* gene, respectively. The total frequency of rare sequence variants in intronic regions was less than 7% (Table S1).

All 117 rare sequence variants were classified as pathogenic or likely pathogenic according to ACMG guidelines, as described in Materials and Methods [21,22] (Tables S1 and S2), with the exception of variants c.2153A>G, c.3563C>G and c.3700G>A, which were classified as “uncertain”, since their clinical significance is unknown or conflicting data are reported. However, during genetic analysis, these variants should not be excluded, at least until additional data clarify their role. 

### 3.2. Phenotype Features of PXE Patients

Of the 310 patients with confirmed clinical and molecular diagnoses, most were female (228), with a female to male ratio of 2.7. The median age of these patients was 40 yrs. (range from 6 to 77 yrs.). Recognition of first clinical manifestation by a physician was at the mean age ± SD of 21 ± 14 yrs. It should be noted that the time required to receive a diagnosis of PXE from its first clinical manifestation was 12 ± 13 yrs. (range from 0.4 months to 50 years). This length of time has been progressively reduced with increased and widespread knowledge of the disease.

Looking at differences between males and females, it should be noted that first manifestations were recognized earlier in females than in males (18 ± 12 yrs. vs. 27 ± 18 yrs.; *p* < 0.001); this is very likely due to the greater attention that women pay to skin alterations, or to an increased susceptibility of female skin to a loss of elasticity. 

**Figure 3:**
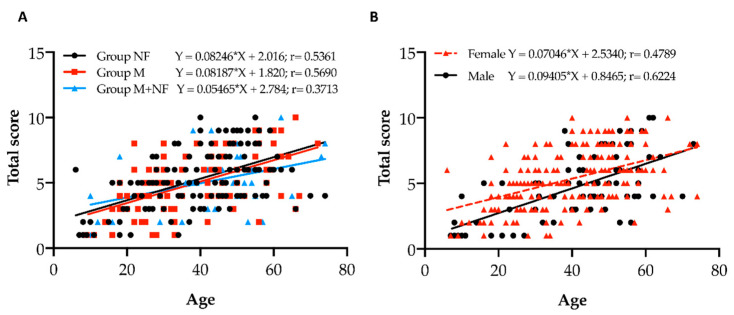
The severity of clinical manifestations, evaluated as total score measured at the time of molecular diagnosis, is significantly dependent on age, with it being higher in aging patients; however, it was not related to either type of variant (**A**) nor to gender (**B**). Group M = two missense variants; group NF = two sequence variants causing reduced protein production or a protein to be likely non-functional; group M + NF = one missense and one “NF” variant.

In the attempt to perform a genotype–phenotype correlation, the 310 patients were divided into three groups according to the characteristics of rare sequence variants: (i) group M (50 patients), which is characterized by two missense variants; (ii) group NF (149 patients), comprising patients exhibiting two sequence variants that cause reduced protein production or a protein to be likely non-functional (i.e., splicing, stop codon, frameshift, deletion, duplication); and (iii) group M+NF (111 patients), comprising patients bearing one missense and one “NF” variant. The severity of the clinical phenotype (total score) was calculated in agreement with the Phenodex index reported by Legrand [19]. After plotting the age and total score for each group, we performed a linear regression analysis. The severity of manifestations increased significantly with age in all three groups (*p*_M_ < 0.0001; *p*_NF_ < 0.0001 and *p*_M+NF_ = 0.0120), but there were no differences among the three fitted lines (*p* = 0.4170) (Figure 3A).

Since clinical manifestations were not related to the type of sequence variant, the severity of the clinical phenotype was analyzed separately in females and in males. We found that the total score was dependent on age but not on gender (*p* = 0.1472) (Figure 3B). The same trend was observed for each score category (data not shown).

The frequency of mitral valve prolapse in mostly asymptomatic PXE patients was 8%, and differed significantly from that of the European population (3.1%; *p* = 0.0064) [54]. No significant differences were observed between female (7%) and male (2%) PXE patients (*p* = 0.2612). 

Since the incidence rate of MI and stroke increases linearly with age, with differences depending on the country [55,56], we compared our data with those of the Italian population at a comparable age (i.e., MI varies from <0.4% before the age of 55 to 4% over the age of 65; stroke varies from <0.2% before the age of 65, and up to 6.5% between 65–84 years of age) [(http://www.salute.gov.it/ (accessed on 25 March 2021))] [57]. In particular, 7/310 PXE patients reported MI. Of these, five patients (1.6%) reported a history of MI before the age of 45 years in the absence of reported cardiovascular risk factors. The incidence was therefore significantly higher if compared to that of the Italian population at the same age (*p* < 0.0001). Moreover, 7/310 PXE patients (2.3%) suffered from ischemic stroke before the age of 65, and this incidence was significantly higher than that of the Italian population (*p* < 0.0001). 

Furthermore, whereas the occurrence of microcalcifications in several organs, including the kidney, was a frequent asymptomatic finding, the incidence of nephrolithiasis in Italian PXE patients was similar to that of the general population (1.7%) [58]. 

### 3.3. Fundoscopic Findings in PXE Patients

**Figure 4:**
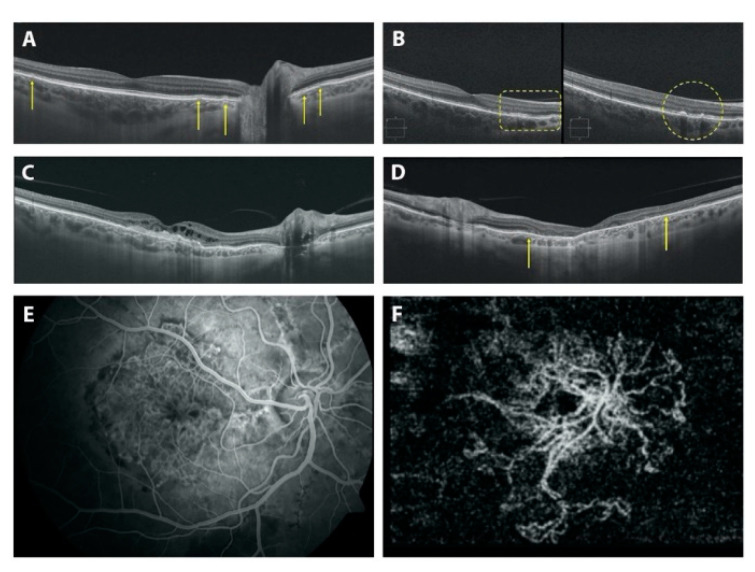
Optical coherence tomography scan showing: (**A**) angioid streaks of different size (yellow arrows); (**B**) retinal pigment epithelium and outer retina layer abnormalities (yellow contour) observed with scan passing at the fovea (left) or passing on dark macular deposits (right); (**C**) atrophy of the retinal pigment epithelium and retinal layers, and hyper-reflective abnormalities due to CNV; (**D**) macular atrophy in a patient affected by pattern dystrophy, with no signs of choroidal neovascularization; the interruptions of the outer nuclear layer are highlighted by the yellow arrows. (**E**) Fluorescein angiography and (**F**) optical coherence tomography angiography showing choroidal neovascularization.

All 310 PXE patients underwent routine ophthalmological examination, OCT and fluorangiography to reveal typical PXE ocular findings (Figure 4). 

According to the Phenodex index, 3% of patients were assigned the E1 score (i.e., *peau d’orange*), 53% were assigned the E2 score (i.e., angioid streaks) and the E3 score was attributable to 44% of patients (i.e., bleeding/scarring). 

**Figure 5:**
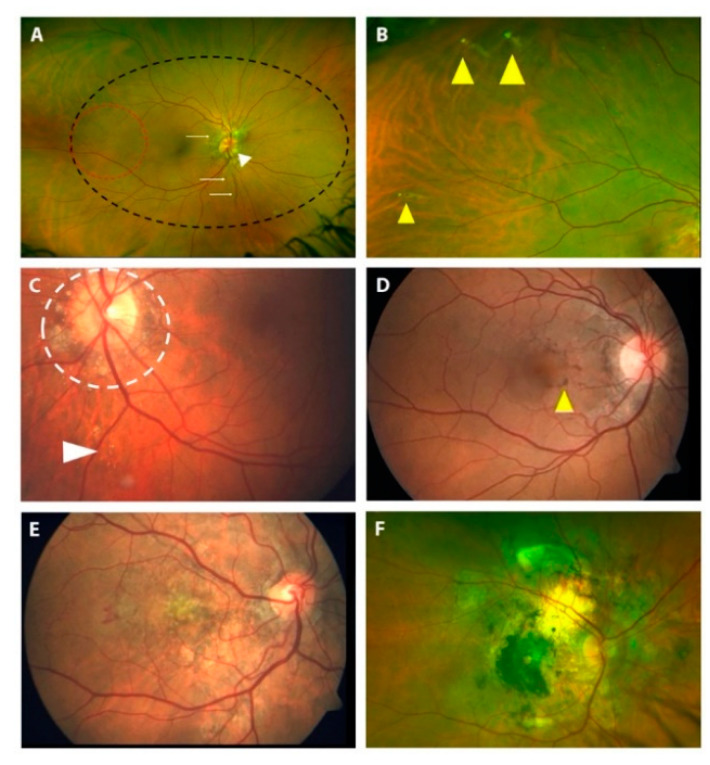
Color fundus photographs showing: (**A**) angioid streaks (white arrows), optic disc drusen (white arrowhead), *peau d’orange* (red circle) and *coquille d’oeuf* (black circle); (**B**) peripheral comet lesions (yellow arrowheads) as small, punctiform, whitish lesions; (**C**) peripapillary comet lesions (white circle) and comet rain (white arrowheads); (**D**) pattern dystrophy-like changes classified as “*fundus pulverulentus*” (yellow arrowhead); (**E**) choroidal neovascularization; (**F**) posterior pole atrophy.

**Figure 6:**
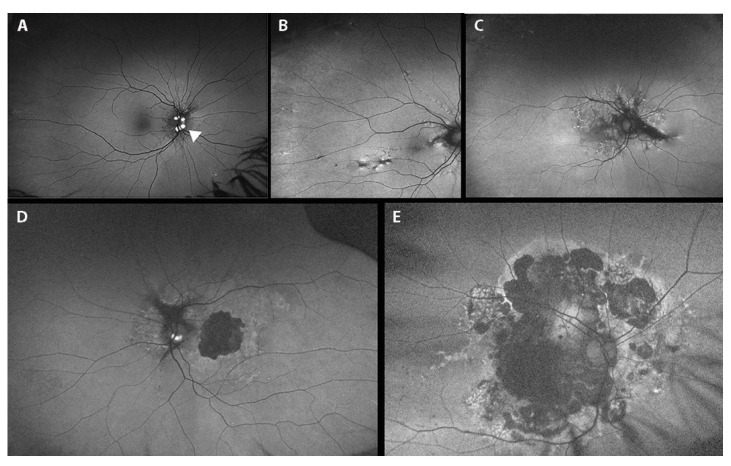
Fundus auto-fluorescence photographs (**A**–**E**) showing: (**A**) optic disk drusen (white arrowheads); (**B**) peripheral comet lesions; (**C**) pattern dystrophy like-changes as hyper/hypo-autofluorescent alterations; (**D**) pattern dystrophy-like changes with macular atrophy (central hypo-autofluorescence alteration); (**E**) posterior pole atrophy.

Of these 310 PXE patients, 53 underwent a further in-depth ophthalmological examination using multimodal fundus imaging approaches; more specifically, we performed wide-field fundus imaging to characterize retinal abnormalities, which are spread all over the fundus (Figure 5 and Figure 6). Ophthalmological features and genetic findings for each of the 53 patients are reported in Tables S3 and S4, respectively. 

In particular, this group of patients was comprised of 9 males (17%) and 44 females (83%), and the mean age ±SD was 47 ± 21 (range 9–69 years) and 43 ± 14 (range 7–68 years), respectively. In particular, 58 eyes of 32 patients (58/106; 55%) presented a visual acuity of 10/10. Eleven eyes of ten patients (11/106; 10.4%) had a visual acuity of <1/10. 

Table 2 shows the ocular manifestations found in 53 PXE patients grouped according to life decade. 

*Peau d’orange* and *coquille d’oeuf* (Figure 5A) are among the first alterations that can be observed in PXE patients (Table 2 and Table S3). Interestingly, *peau d’orange* was not present in 16 eyes of 8 patients (16/106; 15%), while angioid streaks were detectable. On the contrary, in four patients (age 7, 9, 24 and 31yrs.), angioid streaks were not detectable, although *peau d’orange* was present in all of them. If we consider the mean age of patients with *peau d’orange*, in the absence of angioid streaks (mean age 17.8 yrs.; 7–31 yrs.; 10/10 visual acuity of all patients in both eyes), we could note that these patients were younger than the patients with angioid streaks, but without “*peau d’orange*” (59.8 yrs.; 48–69 yrs.).

Angioid streaks (AS) (Figure 4A and Figure 5A) were present in 92.5% of patients but were not detected in younger PXE patients, as reported in other studies [11]; however, they were also present in two patients (24 and 31 years old) who presented only *peau d’orange* or *peau d’orange* and comet lesions, respectively (Table S3). Moreover, in 15 out of 53 patients, AS involved the fovea. We potentially underestimated the real prevalence of foveal AS in PXE patients because, in some cases of CNV, the macular area is severely compromised, so we cannot know the origin of CNV.

Comet lesions (CL) (Figure 5B,C and Figure 6B), which are small, roundish chorioretinal atrophies with focal hyper-pigmentation observed as white bodies in the midperiphery of the fundus, were present in 75.5% of patients (Table 2). In particular, we reported peripapillary CL in 22 eyes of 13 patients (22/106; 21%), and a “comet rain” associated with peripapillary CL in 11 eyes of 8 patients (11/106; 10.4%). 

Pattern dystrophy-like changes (PD) were observed in 41.5% of patients (Table 2 and Table S3), such as *fundus pulverulentus* (Figure 5D and Figure 6C,D), yellowish deposits at the posterior pole, butterfly shaped dystrophy and vitelliform dystrophy. We did not recognize a specific pattern phenotype in all PD. In fact, in the advanced stages of the disease, we were unable to discern a specific pattern due to the presence of retinal atrophy; therefore, we were not able to classify 22 eyes of 12 patients. That said, the recognition of PD signs using the wide-field FAF technique is still important, regardless of specific classification. Interestingly, PD were absent in patients under the age of 30 years; they increased in value by around 25% over the next two decades and were particularly frequent (>70%) in elderly PXE patients (age > 50 years).

Choroidal neovascularization (CNV) was present in 50.9% of patients (Table 2 and Table S3). In particular, 43 eyes of 24 patients and six eyes of four patients presented foveal and extrafoveal CNV, respectively. The mean value of visual acuity was 5.5/10; SD ± 0.67; nlp-10/10). If we consider patients without CNV, they were younger (mean age ± SD was of 34 ± 12 years) than patients affected with CNV (mean age ± SD was of 55 ± 9 years) (Figure 4E,F and Figure 5E).

Atrophy (Figure 4C,D, Figure 5F and Figure 6E,F) was present in 44 eyes of 23 patients (44/106; 41.5%; range 48–69 years) (Table S3). A total of 40 of the 44 eyes (40/44; 91%) presented a history of CNV, while four eyes of three patients developed atrophy in the absence of CNV (without signs or prior therapy for a CNV). Two eyes of two patients presented pattern dystrophy-related changes: *fundus pulverulentus* and vitelliform dystrophy, respectively; the other two eyes belong to a PXE patient affected by diabetic retinopathy which is complicated by diabetic macular edema that is treated using laser therapy (in this patient, we were not able to discern the real contribution of PXE-related dystrophy, diabetic retinopathy or laser treatment to the atrophy). In 10 eyes of 5 patients (10/44; 22.7%), atrophy was present in absence of pattern dystrophy-like changes, and all of them presented CNV. In seven eyes of eight patients (mean age 46.4 yrs. SD ± 17.0), we identified pattern dystrophy-like changes, but no signs of CNV and no signs of atrophy were apparent. Moreover, 24 eyes of 14 patients (24/106; 22.6%) showed an entire posterior pole atrophy.

Optic disk drusen (ODD) was detected in 4.7% of patients (Figure 5A and Figure 6A and Table 2). 

### 3.4. Update of Phenodex Index: Focus on Ocular Manifestations 

**Figure 7:**
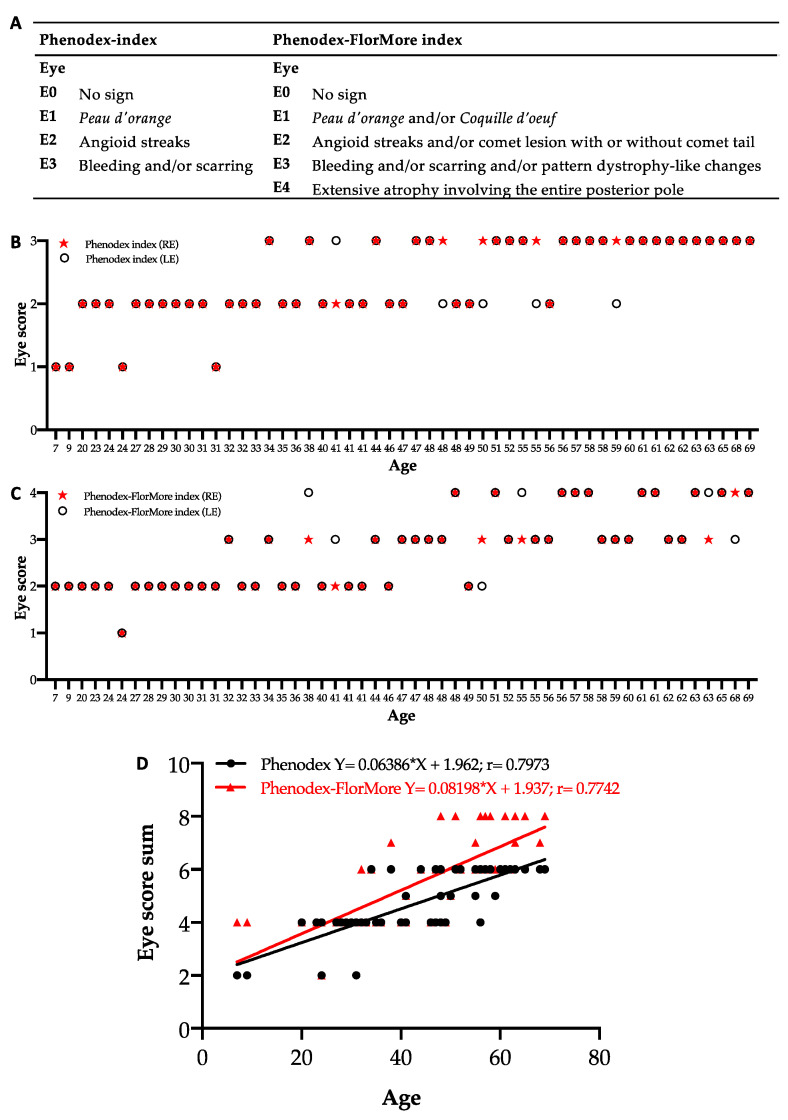
(**A**) Eye score according to the Phenodex or Phenodex-FlorMore index, respectively. (**B**,**C**) Graphs show the right (RE) and left (LE) eye score of each patient according to the Phenodex or Phenodex-FlorMore index, respectively. (**D**) Scatter diagrams and linear regression analysis of the sum of eye scores in PXE patients according to the Phenodex and Phenodex-FlorMore index.

In light of the results from the present study, we implemented the ophthalmologic manifestations reported in the Phenodex index (Figure 7A, left panel) in a newly proposed Phenodex-FlorMore clinical score system, as shown in Figure 7A (right panel). Figure 7B,C show the right and left eye score of each patient calculated according to the Phenodex and Phenodex-FlorMore index, respectively. Although the disease affects both eyes, some patients (i.e., 9% or 11% of patients, depending on the index applied) showed a different score between the right and left eye. The highest score in both eyes was observed at 34 or 48 years, according to Phenodex or Phenodex-FlorMore index, respectively.

To have a global picture of the severity of the disease in each patient, we summed the score assigned to each eye according to the two indexes (Figure 7D). The eye score sum correlates positively with age, independent of the index score system (*p* < 0.0001); however, the two lines show trait divergence towards adulthood. These findings highlight that the Phenodex-FlorMore index improves the stratification of patients in adulthood/old age, where ocular manifestation may be different between the two eyes.

## 4. Discussion

The present study shows data for one of the largest cohorts of PXE patients from the same ethnic background. 

Biomolecular analyses of Italian PXE patients revealed 117 different rare sequence variants distributed on the whole *ABCC6* gene, although some hotspots were present, with differences from other studies depending on geographical areas/ethnicity [59]. It should be noted that the widespread use of databases and bioinformatic platforms (e.g., 1000G, GnomAD) allowed us to reconsider variants previously categorized as potentially pathogenic (e.g., c.346-6G>A; c.1249G>A, p.Val417Met; c.3507-3C>T; c.3803G>A, p.Arg1268Gln) as polymorphisms [60]. Despite the homogeneous ethnicity and the number of patients, which is extremely large for a rare disease, we failed to establish a genotype–phenotype correlation. These data further underline the complexity of the PXE genotype, as rare sequence variants in other genes can also contribute to the pathogenesis of the disease [30,48,61], and/or additional polymorphisms can modulate the onset, the progression and the severity of the disease [27,48,61,62]. Bartstra and coworkers recently described a correlation between the type of variants and specific parameters as calcification mass score and choroidal neovascularization in PXE [63]. This discrepancy is due to the different parameters used to correlate the type of sequence variants, as we have adopted the Phenodex index which, being mainly based on clinical evaluations, has been widely used in the routine clinical practice to easily reflect the global severity of the disease [21,22].

Given the number of patients investigated, we were also able to better evaluate the occurrence of less frequent alterations (e.g., stroke, myocardial infarction, nephrolithiasis).

The present study indicates an incidence of cardio– and cerebral–vascular complications that are higher in PXE compared to age-matched controls; although, these are lower than those reported in other studies [5,6]. The higher incidence that can be observed could be, at least in part, related to the higher frequency of *MTHFR* polymorphisms in Italian PXE patients [40], and it seems to anticipate approximately 1 decade of findings observed in the general population.

As far as the occurrence of nephrolithiasis, values are in contrast to data reported by Legrand and collaborators [19]. Differences may be due to the number of patients under investigation, to genetic background, diet or even to the greater paid attention to a healthy lifestyle generally observed in our patients.

Recent advances in retinal imaging techniques have allowed us to characterize a number of clinical aspects of PXE-related retinopathy, and to consequently stratify these patients in relation to the age-dependent progression of these manifestations. For instance, ultrawide field imaging showed the presence of *coquille d’oeuf*, that is a confluent area of opacity due to widespread infiltration of calcium [64]. This, in turn, revealed the area of the Bruch’s membrane that is already calcified in young PXE patients [65]. Therefore, both *peau d’orange* and *coquille d’oeuf* represent the first consequences of Bruch’s membrane calcification. 

Pattern dystrophy-like changes are also frequently observed in patients with PXE, and these vary, depending on the study, between 10%, 46% and 70% of cases [66,67,68]. Differences in the incidence of PD according to the age of patients indicate that these changes represent a stage in the natural history of the disease [67,68,69,70]. Moreover, pattern dystrophy-like changes and atrophy involved an area which overlapped with the area of *coquille d’oeuf*. Therefore, the retina, in particular where Bruch’s membrane is affected, develops progressive RPE and outer retinal abnormalities (pattern dystrophy-like changes and atrophy) [33,67,68,70]. Early atrophic abnormalities (in patients with pattern dystrophy) appeared as focal areas of a reduced FAF signal, with more or less sharp margins mainly located at the posterior pole, which typically became confluent over time. This multi-lobular pattern, previously reported by other authors [70,71], resembled the “diffuse trickling” pattern described in geographic atrophy in age-related macular degeneration [33]. This pattern was particularly evident in patients with pattern dystrophy without previous CNV; this could be due to the absence of secondary fibrotic/scarring processes, which modified the appearance of retinal abnormalities. In fact, during the disease, the progressive coalesced patches of atrophy formed a circumscribed area of chorioretinal atrophy. In our series, below 35 years of age, nobody presented pattern dystrophy-like changes and/or atrophy. It should be noted that pattern dystrophy can play a role in the development of atrophy in the absence of previous CNV [68,70]. Since atrophy is almost universally observed in older PXE patients, independently from presence or absence of a CNV, it may be seen as the natural endpoint of PXE-associated retinal disease [72,73], and can represent a functionally limiting disease manifestation in PXE [33,74]. Furthermore, late-stage atrophy appeared to involve the entire *coquille d’oeuf* area [33]. For these reasons, the identification of the entire area of *coquille d’oeuf* in PXE patients allows us to identify the fundus area which will become atrophic over time, independent of other PXE complications such as CNV. 

Previous studies have demonstrated a higher incidence of optic disk drusen in PXE patients compared to the normal population [67,75,76]. Although the presence of ODD has been associated with an increased risk of visual field loss due to compression of the unmyelinated retinal ganglion cell axons and surrounding blood vessels [77], a recent study demonstrated no central visual field defects in PXE patients [78]. Therefore, at present, due to the low number of patients presenting ODD in this cohort, we are unable to confirm this latest finding, and additional longitudinal studies are required before assigning a specific pathogenic role to the severity progression of PXE.

In the light of these results, we propose a Phenodex-FlorMore index as a further update of the already modified Phenodex index [19] (Table 3). According to the Phenodex index [19,20], the ocular phenotype is based on the presence of *peau d’orange*, angioid streaks and bleeding and/or scarring, assigning the highest score depending on the most severe manifestation observed in a patient. However, several manifestations are found in PXE patients; although, at present, they are not taken into consideration when an ocular score value is assigned to patients. In particular, *coquille d’oeuf* represents a risk of atrophy over time; comet lesions, with or without a comet tail, are considered the only pathognomonic manifestation of PXE [79], and they have a significant diagnostic value, especially in younger PXE subjects where angioid streaks are not always already developed and detectable [65]. Moreover, as recently suggested [11], patients over 50 may require a different approach to better visualize the severity of the disease and atrophy, and pattern dystrophy-like changes should be taken into consideration to better evaluate disease progression and to improve PXE patients’ counselling in order to enable better decision making in clinical practice.

## Figures and Tables

**Table 1:** Rare sequence variants found in Italian PXE patients. *N* = new rare sequence variant.

Intron/ Exon	Nucleotide Variation	Amino Acid Variation	Ref.	Intron/ Exon	Nucleotide Variation	Amino Acid Variation	Ref.
IVS1	c.36+1dupG	Loss of splice donor site	*N*	21	c.2678C>A	p.Ser893Ter	[19]
IVS1	c.36+1G>C	Loss of splice donor site	[27]	21	c.2728_2746dupTGGATGACCCTGACAGGGC	p.Trp918Ter	[28]
2	c.113G>C	p.Trp38Ser	[29]	IVS21	c.2787+1G>T	Loss of splice donor site	[30]
2	c.117_118insC	p.Met42HisfsTer59	[27]	22	c.2836_2860delinsTCTGCCTCT	p.Leu946SerfsTer18	*N*
2	c.196dupT	p.Ser66PhefsTer35	[31]	22	c.2848G>A	p.Ala950Thr	[20]
5	c.557delT	p.Leu186ArgfsTer46	[28]	22	c.2900G>A	p.Trp967Ter	*N*
6	c.613G>T	p.Glu205Ter	*N*	23	c.3037G>A	p.Gly1013Arg	*N*
IVS7	c.794+1G>A	Loss of splice donor site	*N*	23	c.3088C>T	p.Arg1030Ter	[17]
8	c.913C>T	p.Gln305Ter	[28]	23	c.3109G>A	p.Glu1037Lys	[27]
8	c.940G>A	p.Gly314Arg	[28]	23	c.3142_3144delTTC	p.Phe1048del	[27]
8	c.951C>A	p.Ser317Arg	[32]	24	c.3340C>T	p.Arg1114Cys	[14]
8	c.956T>A	p.Ile319Asn	[33]	24	c.3341G>A	p.Arg1114His	[13]
8	c.960delC	p.Ser321ValfsTer35	[34]	24	c.3380T>C	p.Met1127Thr	[14]
8	c.989delA	p.Lys330SerfsTer26	[28]	24	c.3389C>T	p.Thr1130Met	[35]
9	c.1091C>G	p.Thr364Arg	[31]	24	c.3398G>A	p.Gly1133Asp	[20]
9	c.1132C>T	p.Gln378Ter	[31]	24	c.3412C>T	p.Arg1138Trp	[36]
9	c.1145G>A	p.Arg382Gln	[37]	24	c.3413G>A	p.Arg1138Gln	[36]
9	c.1160G>T	p.Gly387Val	*N*	24	c.3421C>T	p.Arg1141Ter	[12]
9	c.1171A>G	p.Arg391Gly	[38]	24	c.3490C>T	p.Arg1164Ter	[39]
9	c.1174A>G	p.Lys392Glu	*N*	24	c.3491G>A	p.Arg1164Gln	[40]
9	c.1175A>G	p.Lys392Arg	*N*	24	c.3307-940_3506+660del	p.?	[18]
10	c.1220G>A	p.Gly407Asp	*N*	25	c.3542G>A	p.Gly1181Asp	[41]
10	c.1220G>T	p.Gly407Val	[28]	25	c.3544dupC	p.Leu1182ProfsTer96	*N*
10	c.1255C>T	p.Arg419Trp	*N*	25	c.3563C>G	p.Thr1188Arg	*N*
10	c.1256G>A	p.Arg419Gln	[42]	26	c.3661C>T	p.Arg1221Cys	[43]
10	c.1284C>G	p.Asn428Lys	[19]	26	c.3662G>A	p.Arg1221His	[20]
10	c.1308G>A	p.Trp436Ter	[28]	26	c.3677T>C	p.Leu1226Pro	[33]
10	c.1318T>G	p.Cys440Gly	[14]	26	c.3700G>A	p.Glu1234Lys	[44]
12	c.1484T>A	p.Leu495His	[32]	26	c.3712G>T	p.Asp1238Tyr	[38]
12	c.1526C>G	p.Ala509Gly	[19]	26	c.3735G>A	p.Glu1245=	*N*
12	c.1552C>T	p.Arg518Ter	[34]	IVS26	c.3736-1G>A	Loss of splice acceptor site	[36]
12	c.1553G>A	p.Arg518Gln	[45]	27	c.3774_3775insC	p.Trp1259LeufsTer19	[20]
IVS13	c.1779+1G>C	Loss of splice donor site	[28]	27	c.3823C>T	p.Arg1275Ter	[14]
14	c.1798C>T	p.Arg600Cys	[14]	27	c.3871delG	p.Ala1291GlnfsTer68	[28]
14	c.1799G>A	p.Arg600His	[41]	27	c.3880_3882delAAG	p.Lys1294del	[20]
14	c.1857dupC	p.Ser620LeufsTer121	[20]	28	c.3892G>A	p.Val1298Ile	*N*
16	c.1961C>T	p.Pro654Leu	[37]	28	c.3902C>T	p.Thr1301Ile	[17]
16	c.1987G>A	p.Gly663Ser	[46]	28	c.3904G>A	p.Gly1302Arg	[17]
16	c.1987G>T	p.Gly663Cys	[20]	28	c.3940C>T	p.Arg1314Trp	[12]
16	c.1999delG	p.Ala667GlnfsTer21	[20]	28	c.3989T>C	p.Ile1330Thr	[37]
17	c.2018T>C	p.Leu673Pro	[17]	29	c.4015C>T	p.Arg1339Cys	[39]
17	c.2093A>C	p.Gln698Pro	[20]	29	c.4036C>T	p.Pro1346Ser	[14]
17	c.2095G>A	p.Glu699Lys	[37]	29	c.4041G>A	p.Gln1347=	[28]
17	c.2153C>A	p.Asp718Gly	[47]	29	c.4055T>C	p.Phe1352Ser	[48]
IVS17	c.2248-2_2248-1delAG	Loss of splice acceptor site	[49]	29	c.4070G>C	p.Arg1357Pro	[28]
IVS17	c.2247+1G>A	Loss of splice donor site	[27]	29	c.4159_4171dupCTGCCCGGCCAGC	p.Leu1391ProfsTer10	*N*
18	c.2263G>A	p.Gly755Arg	[20]	29	c.4182delG	p.Lys1394AsnfsTer9	[13]
18	c.2264G>A	p.Gly755Glu	[27]	29	c.4198G>A	p.Glu1400Lys	[38]
18	c.2266G>A	p.Gly756Ser	[28]	IVS29	c.4208+1G>A	Loss of splice donor site	[28]
18	c.2278C>T	p.Arg760Trp	[50]	30	c.4318delA	p.Met1440CysfsTer24	[14]
18	c.2294G>A	p.Arg765Gln	[17]	30	c.4361T>C	p.Leu1454Pro	*N*
18	c.2307_2308insA	p.Ala771GlyfsTer8	*N*	30	c.4403G>A	p.Arg1468Gln	*N*
18	c.2329G>A	p.Asp777Asn	[20]	30	c.del30	p.?	[51]
18	c.2383G>T	p.Val795Phe	[27]	1_31	c.1_4511del	p.?	[52]
19	c.2419C>T	p.Arg807Trp	[32]	11_18	c.dup11-18	p.?	*N*
19	c.2428G>A	p.Val810Met	[14]	23_29	c.2996_4208del	p.?	[17]
19	c.2458G>C	p.Ala820Pro	[33]	24_27	c.3307-1006_3735+1582del	p.?	[18]
19	c.2477T>C	p.Leu826Pro	[53]	25_27	c.3507_3882del	p.?	[49]
19	c.2504G>A	p.Gly835Asp	*N*				

**Table 2:** Ocular manifestations found in PXE patients (pt) in different age groups.

Age	N. of Pt	Pt with PO	%/ Decade	Pt with CO	%/ Decade	Pt with AS	%/ Decade	Pt with CL	%/ Decade	Pt with CNV	%/ Decade	Pt with PD	%/ Decade	Pt with PPA	%/ Decade	Pt with ODD	%/ Decade
<10	2	2	100	2	100	0	0	2	100	0	0	0	0	0	0	0	0
20–29	7	7	100	7	100	6	85.7	5	71.4	0	0	0	0	0	0	1	14.3
30–39	11	11	100	11	100	10	90.9	9	81.8	2	18.2	3	27.3	1	9.1	0	0
40–49	12	11	91.7	11	91.7	12	100	10	83.3	5	41.7	3	25.0	1	8.3	1	8.3
50–59	11	10	90.9	10	90.9	11	100	7	63.6	10	90.9	8	72.7	5	45.5	1	9.1
60–69	10	4	40	4	40	10	100	7	70	10	100	8	80	7	70	0	0
Sum	53	45		45		49		40		27		22		14		3	
Mean			84.9		84.9		92.5		75.5		50.9		41.5		26.4		5.7

PO = *peau d’orange*; CO = *coquille d’oeuf;* AS = angioid streaks; CL = comet lesion; CNV = choroidal neovascularization; PD = pattern dystrophy; PPA = pole posterior atrophy; ODD = optic disk drusen.

**Table 3:** Phenodex-FlorMore scoring system for the evaluation of PXE clinical manifestations.

Organ System Findings	
**Skin**	
S0	No sign
S1	Papules/bumps
S2	Plaques of coalesced papules
S3	Lax and redundant skin
**Eyes (score of RE + score of LE)**	
E0	No sign
E1	*Peau d’orange and/or **Coquille d’oeuf***
E2	Angioid streaks and/or **comet lesion with or without comet tail**
E3	Bleeding and/or scarring and/or **pattern dystrophy-like changes**
**E4**	**Extensive atrophic area involving the entire posterior pole**
**Gastrointestinal**	
G0	No sign
G1	Gastrointestinal bleeding as related to PXE
**Vascular**	
V0	No sign
V1	Weak or absent pulse or peripheral artery disease revealed by vascular imaging
V2	*Intermittent claudication*
V3	Vascular surgery or stroke/transient ischaemic attack (TIA)
**Cardiac**	
C0	No sign
C1	Chest pain/angina/abnormal EKG or abnormal stress test with no symptom or mitral insufficiency
C2	Heart attack
**Renal**	
R0	No sign
R1	Nephrolithiasis
In bold, the features are added. RE= right eye; LE= left eye; EKG= electrocardiogram.	

## Data Availability

Not applicable.

## Supplementary Materials

The following are available online at https://www.mdpi.com/article/10.3390/jcm10122710/s1, Table S1: Rare sequence variants found in Italian PXE patients and classified in according to American College of Medical Genetics and Genomics and Association for Molecular Pathology (ACMG) using VarSome platform, Table S2: Missense point variants evaluated for their pathogenicity using different *in silico* tools, Table S3: Ocular manifestations in PXE patients, Table S4: Frequency of singular ocular manifestations according to the characteristics of rare sequence variants detected in 53 PXE patients who underwent in-depth ophthalmological investigation.
