# Supplementary material for: Phenotypic Features and Genetic Findings in a Cohort of Italian Pseudoxanthoma Elasticum Patients and Update of the Ophthalmologic Evaluation Score
Source: J Clin Med. 2021 Jun 19;10(12):2710. doi: 10.3390/jcm10122710 (PMC8235548; doi:10.3390/jcm10122710)
Supplement: Supplementary file 1 [file jcm-10-02710-s001.zip › Table S1.pdf]

Table S1. Rare sequence variants found in Italian PXE patients and classified in according to American College of Medical Genetics and Genomics and Association for Molecular Pathology (ACMG) using VarSome platform. dbSNP= single nucleotide polymorphism database; N= new rare sequence variant; F= rare sequence variant frequency in relation to the other sequence variants detected within the Italian PXE patient cohort.

Intron / Exon	Nucleotide variation	Amino acid variation	F (%)	dbSNP	Class ACMG
IVS1	c.36+1dupG	Loss of splice donor site	0,188	/	Likely pathogenic
IVS1	c.36+1G>C	Loss of splice donor site	0,942	/	Pathogenic
2	c.113G>C	p.Trp38Ser	0,753	rs72653752	Likely pathogenic
2	c.117_118insC	p.Met42HisfsTer59	0,188	rs746531177	Pathogenic
2	c.196dupT	p.Ser66PhefsTer35	0,753	rs1064793538	Pathogenic
5	c.557delT	p.Leu186ArgfsTer46	0,753	rs1342109356	Pathogenic
6	c.613G>T	p.Glu205Ter	0,377	/	Pathogenic
IVS7	c.794+1G>A	Loss of splice donor site	0,565	rs775749515	Pathogenic
8	c.913C>T	p.Gln305Ter	0,377	rs746625905	Pathogenic
8	c.940G>A	p.Gly314Arg	0,565	/	Likely pathogenic
8	c.951C>A	p.Ser317Arg	0,942	rs78678589	Pathogenic
8	c.956T>A	p.Ile319Asn	0,188	/	Likely pathogenic
8	c.960delC	p.Ser321ValfsTer35	0,188	rs72664226	Pathogenic
8	c.989delA	p.Lys330SerfsTer26	0,377	/	Pathogenic
9	c.1091C>G	p.Thr364Arg	0,565	rs72653759	Likely pathogenic
9	c.1132C>T	p.Gln378Ter	4,896	rs72650699	Pathogenic
9	c.1145G>A	p.Arg382Gln	0,188	rs776373779	Likely pathogenic
9	c.1160G>T	p.Gly387Val	0,188	rs771256512	Likely pathogenic
9	c.1171A>G	p.Arg391Gly	2,260	rs72653762	Pathogenic
9	c.1174A>G	p.Lys392Glu	0,188	rs756498547	Pathogenic
9	c.1175A>G	p.Lys392Arg	0,188	rs748562202	Pathogenic
10	c.1220G>A	p.Gly407Asp	0,188	rs866947308	Likely pathogenic
10	c.1220G>T	p.Gly407Val	0,188	/	Likely pathogenic
10	c.1255C>T	p.Arg419Trp	0,188	rs775853778	Likely pathogenic
10	c.1256G>A	p.Arg419Gln	0,188	rs772434460	Pathogenic
10	c.1284C>G	p.Asn428Lys	0,188	/	Likely pathogenic
10	c.1308G>A	p.Trp436Ter	0,565	/	Pathogenic
10	c.1318T>G	p.Cys440Gly	0,188	rs72653766	Likely pathogenic
12	c.1484T>A	p.Leu495His	0,377	rs72653769	Likely pathogenic
12	c.1526C>G	p.Ala509Gly	0,188	rs779408186	Likely pathogenic
12	c.1552C>T	p.Arg518Ter	6,403	rs72650700	Pathogenic
12	c.1553G>A	p.Arg518Gln	6,026	rs72653772	Pathogenic
IVS13	c.1779+1G>C	Loss of splice donor site	0,188	rs768037422	Pathogenic
14	c.1798C>T	p.Arg600Cys	0,377	rs72653777	Likely pathogenic
14	c.1799G>A	p.Arg600His	0,188	rs761433545	Likely pathogenic
14	c.1857dupC	p.Ser620LeufsTer121	0,565	rs72664218	Pathogenic
16	c.1961C>T	p.Pro654Leu	0,188	rs754695089	Likely pathogenic
16	c.1987G>A	p.Gly663Ser	0,188	rs72653780	Likely pathogenic
16	c.1987G>T	p.Gly663Cys	0,188	rs72653780	Likely pathogenic
16	c.1999delG	p.Ala667GlnfsTer21	2,448	rs72664227	Pathogenic
17	c.2018T>C	p.Leu673Pro	0,188	rs67470842	Likely pathogenic
17	c.2093A>C	p.Gln698Pro	0,188	rs72653783	Likely pathogenic
17	c.2095G>A	p.Glu699Lys	0,188	rs1445084199	Likely pathogenic
17	c.2153C>A	p.Asp718Gly	0,188	rs1357894483	Uncertain significance
IVS17	c.2248-2_2248-1delAG	Loss of splice acceptor site	0,942	rs111113624	Pathogenic
IVS17	c.2247+1G>A	Loss of splice donor site	0,565	rs781190396	Pathogenic
18	c.2263G>A	p.Gly755Arg	0,377	rs72653787	Pathogenic

18	c.2264G>A	p.Gly755Glu	0,188	/	Likely pathogenic
18	c.2266G>A	p.Gly756Ser	0,377	rs1022850449	Likely pathogenic
18	c.2278C>T	p.Arg760Trp	0,942	rs72653788	Likely pathogenic
18	c.2294G>A	p.Arg765Gln	0,942	rs67561842	Pathogenic
18	c.2307_2308insA	p.Ala771GlyfsTer8	0,188	/	Pathogenic
18	c.2329G>A	p.Asp777Asn	0,753	rs72653790	Likely pathogenic
18	c.2383G>T	p.Val795Phe	0,377	/	Likely pathogenic
19	c.2419C>T	p.Arg807Trp	1,130	rs72653793	Likely pathogenic
19	c.2428G>A	p.Val810Met	0,188	rs72653795	Likely pathogenic
19	c.2458G>C	p.Ala820Pro	0,188	rs72653797	Likely pathogenic
19	c.2477T>C	p.Leu826Pro	0,188	rs72653798	Likely pathogenic
19	c.2504G>A	p.Gly835Asp	0,188	rs199990104	Likely pathogenic
21	c.2678C>A	p.Ser893Ter	0,377	rs1481200467	Likely pathogenic
21	c.2728_2746dupTGGATGACCCTGACAGGGC	p.Trp918Ter	0,188	/	Pathogenic
IVS21	c.2787+1G>T	Loss of splice donor site	0,753	rs72664209	Pathogenic
22	c.2836_2860delinsTCTGCCTCT	p.Leu946SerfsTer18	0,188	/	Pathogenic
22	c.2848G>A	p.Ala950Thr	0,188	rs72657689	Likely pathogenic
22	c.2900G>A	p.Trp967Ter	0,188	/	Pathogenic
23	c.3037G>A	p.Gly1013Arg	0,188	/	Likely pathogenic
23	c.3088C>T	p.Arg1030Ter	1,695	rs72653705	Pathogenic
23	c.3109G>A	p.Glu1037Lys	0,188	rs754074990	Likely pathogenic
23	c.3142_3144delTTC	p.Phe1048del	0,188	rs769437554	Likely pathogenic
24	c.3340C>T	p.Arg1114Cys	1,695	rs63749794	Likely pathogenic
24	c.3341G>A	p.Arg1114His	0,188	rs63750427	Likely pathogenic
24	c.3380T>C	p.Met1127Thr	0,377	rs63749998	Likely pathogenic
24	c.3389C>T	p.Thr1130Met	0,188	rs63750459	Likely pathogenic
24	c.3398G>A	p.Gly1133Asp	0,188	/	Likely pathogenic
24	c.3412C>T	p.Arg1138Trp	0,753	rs28939701	Pathogenic
24	c.3413G>A	p.Arg1138Gln	1,507	rs60791294	Likely pathogenic
24	c.3421C>T	p.Arg1141Ter	24,294	rs72653706	Pathogenic
24	c.3490C>T	p.Arg1164Ter	1,507	rs72653744	Pathogenic
24	c.3491G>A	p.Arg1164Gln	0,377	rs63750457	Likely pathogenic
24	c.3307-940_3506+660del	p.?	0,188	/	Pathogenic
25	c.3542G>A	p.Gly1181Asp	0,188	rs114928628	Likely pathogenic
25	c.3544dupC	p.Leu1182ProfsTer96	0,188	rs1555508630	Pathogenic
25	c.3563C>G	p.Thr1188Arg	0,188	/	Uncertain significance
26	c.3661C>T	p.Arg1221Cys	0,565	rs63751215	Likely pathogenic
26	c.3662G>A	p.Arg1221His	0,377	rs63751001	Likely pathogenic
26	c.3677T>C	p.Leu1226Pro	0,377	rs770483331	Likely pathogenic
26	c.3700G>A	p.Glu1234Lys	0,188	/	Uncertain significance
26	c.3712G>T	p.Asp1238Tyr	0,565	/	Likely pathogenic
26	c.3735G>A	p.Glu1245=	0,188	rs281865557	Likely pathogenic
IVS26	c.3736-1G>A	Loss of splice acceptor site	2,448	rs63750273	Pathogenic
27	c.3774_3775insC	p.Trp1259LeufsTer19	1,130	rs72664220	Pathogenic
27	c.3823C>T	p.Arg1275Ter	0,188	rs72653749	Pathogenic
27	c.3871delG	p.Ala1291GlnfsTer68	0,377	/	Likely pathogenic
27	c.3880_3882delAAG	p.Lys1294del	0,565	rs72664235	Likely pathogenic
28	c.3892G>A	p.Val1298Ile	0,188	rs63751325	Likely pathogenic
28	c.3902C>T	p.Thr1301Ile	0,188	rs63750494	Likely pathogenic
28	c.3904G>A	p.Gly1302Arg	0,188	rs63749856	Pathogenic
28	c.3940C>T	p.Arg1314Trp	0,565	rs63750759	Likely pathogenic
28	c.3989T>C	p.Ile1330Thr	0,188	rs760794410	Likely pathogenic
29	c.4015C>T	p.Arg1339Cys	0,565	rs28939702	Likely pathogenic
29	c.4036C>T	p.Pro1346Ser	0,188	rs63751112	Pathogenic

29	c.4041G>A	p.Gln1347=	0,188	rs63751111	Likely pathogenic
29	c.4055T>C	p.Phe1352Ser	0,188	/	Likely pathogenic
29	c.4070G>C	p.Arg1357Pro	0,188	/	Likely pathogenic
29	c.4159_4171dupCTGCCCGGCCAGC	p.Leu1391ProfsTer10	0,377	rs747334212	Pathogenic
29	c.4182delG	p.Lys1394AsnfsTer9	0,753	rs67791546	Pathogenic
29	c.4198G>A	p.Glu1400Lys	1,318	rs63751241	Pathogenic
IVS29	c.4208+1G>A	Loss of splice donor site	0,188	rs1481973160	Pathogenic
30	c.4318delA	p.Met1440CysfsTer24	0,377	rs72664238	Pathogenic
30	c.4361T>C	p.Leu1454Pro	0,188	/	Likely pathogenic
30	c.4403G>A	p.Arg1468Gln	0,188	rs761098006	Pathogenic
30	c.del30	p.?	0,377	/	Pathogenic
1_31	c.1_4511del	p.?	0,942	/	Pathogenic
11_18	c.dup11-18	p.?	0,188	/	Pathogenic
23_29	c.2996_4208del	p.?	5,838	/	Pathogenic
24_27	c.3307-1006_3735+1582del	p.?	0,753	/	Pathogenic
25_27	c.3507_3882del	p.?	0,377	/	Pathogenic
