# Supplementary material for: Phenotypic Features and Genetic Findings in a Cohort of Italian Pseudoxanthoma Elasticum Patients and Update of the Ophthalmologic Evaluation Score
Source: J Clin Med. 2021 Jun 19;10(12):2710. doi: 10.3390/jcm10122710 (PMC8235548; doi:10.3390/jcm10122710)
Supplement: Supplementary file 1 [file jcm-10-02710-s001.zip › Table S2.pdf]

Table S1. Missense point variants evaluated for their pathogenicity using different *in silico* tools.

Exon	Nucleotide variation	Amino acid variation	PolyPhen-2	Panther	Align GVGD
9	c.1160G>T	p.Gly387Val	Probably damaging (Score 1.00)	Probably damaging (PT 456)	Class C65
9	c.1174A>G	p.Lys392Glu	Probably damaging (Score 1.00)	Probably damaging (PT 842)	Class C55
9	c.1175A>G	p.Lys392Arg	Probably damaging (Score 1.00)	Probably damaging (PT 842)	Class C25
10	c.1220G>A	p.Gly407Asp	Probably damaging (Score 1.00)	Probably damaging (PT 910)	Class C65
10	c.1255C>T	p.Arg419Trp	Probably damaging (Score 0.983)	Probably damaging (PT 910)	Class C65
19	c.2504G>A	p.Gly835Asp	Probably damaging (Score 0.999)	Probably damaging (PT 910)	Class C65
23	c.3037G>A	p.Gly1013Arg	Probably damaging (Score 0.999)	Probably damaging (PT 221)	Class C65
25	c.3563C>G	p.Thr1188Arg	Probably damaging (Score 0.965)	Probably benign (PT 97)	Class C65
26	c.3735G>A	p.Glu1245=	/	Probably damaging (PT 842)	/
28	c.3892G>A	p.Val1298Ile	Probably damaging (Score 0.965)	Probably damaging (PT 842)	Class C25
30	c.4361T>C	p.Leu1454Pro	Probably damaging (Score 0.99)	Probably bening (PT 176)	Class C65
30	c.4403G>A	p.Arg1468Gln	Probably damaging (Score 0.976)	Probably damaging (PT 456)	Class C35

PT: preservation time

FI: Functional impact
