# Supplementary material for: Phenotypic Features and Genetic Findings in a Cohort of Italian Pseudoxanthoma Elasticum Patients and Update of the Ophthalmologic Evaluation Score
Source: J Clin Med. 2021 Jun 19;10(12):2710. doi: 10.3390/jcm10122710 (PMC8235548; doi:10.3390/jcm10122710)
Supplement: Supplementary file 1 [file jcm-10-02710-s001.zip › Table S3.pdf]

Table S3. Ocular manifestations in PXE patients.

ID	Age	Sex	Eye	Visual acuity (Snellen)	EqS	Anterior segment	PO	CO	AS	Foveal AS	Peripheral CL	Peripapillary CL	CNV	PD like changes	Atrophy	Disc drusen
1	7	F	RE	10/10	0.00	N	p	p	nd	nd	p	nd	nd	nd	nd	nd
			LE	10/10	0.00	N	p	p	nd	nd	p	nd	nd	nd	nd	nd
2	9	M	RE	10/10	-0.63	N	p	p	nd	nd	p	nd	nd	nd	nd	nd
			LE	10/10	-1.75	N	p	p	nd	nd	p	nd	nd	nd	nd	nd
3	20	M	RE	10/10	0.00	N	p	p	p	nd	p	p	nd	nd	nd	nd
			LE	10/10	0.00	N	p	p	p	nd	p	p	nd	nd	nd	nd
4	23	F	RE	10/10	0.00	N	p	p	p	p	p	p	nd	nd	nd	nd
			LE	10/10	0.00	N	p	p	p	p	p	p	nd	nd	nd	nd
5	24	F	RE	10/10	0.00	N	p	p	p	nd	p	p	nd	nd	nd	nd
			LE	10/10	0.00	N	p	p	p	nd	p	p	nd	nd	nd	nd
6	24	F	RE	10/10	0.00	N	p	p	nd	nd	nd	nd	nd	nd	nd	nd
			LE	10/10	0.00	N	p	p	nd	nd	nd	nd	nd	nd	nd	nd
7	27	F	RE	10/10	0.00	N	p	p	p	nd	p	nd	nd	nd	nd	nd
			LE	10/10	0.00	N	p	p	p	p	p	p	nd	nd	nd	nd
8	28	F	RE	10/10	-2.75	N	p	p	p	p	nd	nd	nd	nd	nd	nd
			LE	10/10	-2.75	N	p	p	p	p	nd	nd	nd	nd	nd	nd
9	29	F	RE	10/10	-5.25	N	p	p	p	nd	p	nd	nd	nd	nd	p
			LE	10/10	-3.75	N	p	p	p	nd	p	nd	nd	nd	nd	p
10	30	F	RE	10/10	0.00	N	p	p	p	nd	p	nd	nd	nd	nd	nd
			LE	10/10	0.00	N	p	p	p	nd	nd	nd	nd	nd	nd	nd
11	30	F	RE	10/10	0.00	N	p	p	p	nd	p	p	nd	nd	nd	nd
			LE	10/10	0.00	N	p	p	p	nd	p	p	nd	nd	nd	nd
12	31	F	RE	10/10	0.00	N	p	p	p	nd	p	nd	nd	nd	nd	nd
			LE	10/10	0.00	N	p	p	p	nd	p	nd	nd	nd	nd	nd
13	31	F	RE	10/10	0.00	N	p	p	nd	nd	p	nd	nd	nd	nd	nd
			LE	10/10	0.00	N	p	p	nd	nd	p	nd	nd	nd	nd	nd
14	32	F	RE	10/10	0.00	N	p	p	p	nd	nd	p	nd	FP	nd	nd
			LE	9/10	0.00	N	p	p	p	nd	p	p	nd	FP	nd	nd
15	32	F	RE	10/10	-4.25	N	p	p	p	nd	nd	nd	nd	nd	nd	nd
			LE	10/10	-4.50	N	p	p	p	nd	nd	nd	nd	nd	nd	nd
16	33	F	RE	10/10	0.00	N	p	p	p	nd	p	nd	nd	nd	nd	nd
			LE	10/10	0.00	N	p	p	p	nd	p	nd	nd	nd	nd	nd
17	34	F	RE	5/10	-1.25	N	p	p	p	p	p	nd	foveal	BD	nd	nd
			LE	3.2/10	-1.50	N	p	p	p	p	p	nd	foveal	BD	nd	nd
18	35	F	RE	10/10	0.00	N	p	p	p	p	p	nd	nd	nd	nd	nd
			LE	10/10	0.00	N	p	p	p	p	p	nd	nd	nd	nd	nd
19	36	F	RE	10/10	1.88	N	p	p	p	nd	nd	nd	nd	nd	nd	nd
			LE	10/10	1.50	N	p	p	p	nd	nd	nd	nd	nd	nd	nd
20	38	F	RE	10/10	0.00	N	p	p	p	nd	p	nd	foveal	FP	nd	nd
			LE	1.6/10	0.00	N	p	p	p	nd	p	nd	foveal	UN	posterior pole atrophy	nd
21	40	F	RE	10/10	0.00	N	p	p	p	p	p	nd	nd	nd	nd	nd
			LE	10/10	0.00	N	p	p	p	nd	p	nd	nd	nd	nd	nd
22	41	M	RE	2.5/10	0.00	N	p	p	p	nd	p	nd	nd	nd	nd	nd
			LE	10/10	0.00	N	p	p	p	p	p	nd	foveal	nd	nd	nd
23	41	F	RE	10/10	-4.25	N	p	p	p	nd	p	p	nd	nd	nd	nd
			LE	10/10	-5.00	N	p	p	p	nd	p	p	nd	nd	nd	nd
24	41	F	RE	10/10	0.00	N	p	p	p	nd	p	nd	nd	nd	nd	nd
			LE	10/10	0.00	N	p	p	p	nd	p	nd	nd	nd	nd	nd
25	44	F	RE	10/10	0.00	N	p	p	p	nd	p	nd	extrafoveal	nd	nd	nd
			LE	10/10	0.00	N	p	p	p	nd	p	nd	extrafoveal	nd	nd	nd
26	46	F	RE	10/10	0.00	N	p	p	p	nd	p	nd	nd	nd	nd	nd
			LE	10/10	0.00	N	p	p	p	nd	p	nd	nd	nd	nd	nd
27	47	F	RE	10/10	-3.75	N	p	p	p	p	p	p	nd	FF/YD	nd	nd
			LE	10/10	-3.00	N	p	p	p	p	p	nd	nd	FF/YD	nd	nd
28	47	M	RE	5/10	0.00	N	p	p	p	p	p	nd	foveal	FF/YD	inside the vascular arcades	nd
			LE	4/10	0.00	N	p	p	p	p	p	p	foveal	FF/YD	inside the vascular arcades	nd
29	48	F	RE	2/10	1.50	congenital cataract	p	p	p	nd	nd	nd	foveal	nd	inside the vascular arcades	p
			LE	8/10	0.50	congenital cataract	p	p	p	nd	nd	nd	foveal	nd	inside the vascular arcades	nd
30	48	F	RE	2/10	-6.13	N	p	p	p	p	p	nd	foveal	VD	inside the vascular arcades	nd

			LE	7/10	-7.25	N	p	p	p	p	p	nd	nd	VD	inside the vascular arcades	nd
31	48	F	RE	1.6/10	-0.75	pseudophakic	nd	nd	p	nd	nd	nd	nd	nd	posterior pole atrophy	nd
			LE	1/10	-1.25	cataract	nd	nd	p	nd	nd	nd	nd	nd	posterior pole atrophy	nd
32	49	F	RE	6.2/10	5.50	N	p	p	p	p	p	nd	nd	nd	nd	nd
			LE	1.6/10	6.50	cataract	p	p	p	nd	p	nd	nd	nd	nd	nd
33	50	F	RE	10/10	6.75	N	p	p	p	p	p	p	extrafoveal	nd	nd	nd
			LE	10/10	5.50	N	p	p	p	nd	p	p	nd	nd	nd	nd
34	51	M	RE	1.6/10	-0.75	N	nd	nd	p	nd	nd	nd	foveal	UN	posterior pole atrophy	nd
			LE	1/16	0.00	N	nd	nd	p	nd	nd	nd	foveal	UN	posterior pole atrophy	nd
35	52	F	RE	1/20	0.00	N	p	p	p	p	p	nd	foveal	nd	inside the vascular arcades	nd
			LE	light perception	0.00	N	p	p	p	p	p	nd	foveal	nd	inside the vascular arcades	nd
36	55	F	RE	6.2/10	0.00	N	p	p	p	nd	p	p	foveal	nd	inside the vascular arcades	nd
			LE	hand motion	0.00	N	p	p	p	nd	p	p	foveal	nd	posterior pole atrophy	nd
37	55	F	RE	10/10	-2.50	N	p	p	p	p	nd	nd	foveal	UN	nd	nd
			LE	10/10	-0.75	N	p	p	p	p	nd	nd	nd	UN	nd	nd
38	56	F	RE	10/10	-1.25	N	p	p	p	p	nd	nd	nd	FP	nd	nd
			LE	10/10	-1.50	N	p	p	p	nd	nd	nd	nd	FP	nd	nd
39	56	F	RE	1/20	-3.00	N	p	p	p	nd	p	nd	foveal	UN	posterior pole atrophy	nd
			LE	4/10	-3.50	N	p	p	p	nd	p	nd	foveal	UN	posterior pole atrophy	nd
40	57	F	RE	1.6/10	0.75	N	p	p	p	nd	nd	nd	foveal	UN	posterior pole atrophy	nd
			LE	1.6/10	0.75	N	p	p	p	nd	nd	p	foveal	UN	posterior pole atrophy	nd
41	58	F	RE	1/20	0.00	cataract	p	p	p	nd	p	p	foveal	FF/YD	posterior pole atrophy	nd
			LE	2/10	0.00	psedophakic	p	p	p	nd	p	p	foveal	FF/YD	posterior pole atrophy	nd
42	58	F	RE	10/10	0.00	N	p	p	p	nd	nd	nd	extrafoveal	UN	nd	nd
			LE	7/10	0.00	N	p	p	p	nd	nd	nd	foveal	UN	inside the vascular arcades	nd
43	59	F	RE	1.6/10	1.00	N	p	p	p	nd	p	nd	foveal	FF/YD	inside the vascular arcades	p
			LE	6.2/10	0.50	N	p	p	p	nd	p	nd	nd	FF/YD	inside the vascular arcades	p
44	60	F	RE	5/10	0.88	cataract	nd	nd	p	nd	nd	nd	foveal	nd	inside the vascular arcades	nd
			LE	1/20	0.00	cataract	nd	nd	p	nd	nd	nd	foveal	nd	inside the vascular arcades	nd
45	61	F	RE	no light perception*	0.00	cataract	nd	nd	p	nd	p	nd	foveal	UN	posterior pole atrophy	nd
			LE	3.2/10	-3.50	cataract	nd	nd	p	nd	p	nd	foveal	UN	posterior pole atrophy	nd
46	61	M	RE	1/10	-1.50	N	nd	nd	p	nd	p	nd	foveal	UN	posterior pole atrophy	nd
			LE	1.6/10	-1.50	N	nd	nd	p	nd	p	nd	foveal	UN	posterior pole atrophy	nd
47	62	M	RE	10/10	-2.00	N	p	p	p	nd	p	nd	foveal	FP	inside the vascular arcades	nd
			LE	2/10	-2.40	N	p	p	p	nd	p	nd	foveal	UN	inside the vascular arcades	nd
48	62	F	RE	1/12	0.00	N	p	p	p	nd	p	nd	extrafoveal	FF/YD	inside the vascular arcades	nd
			LE	10/10	0.00	N	p	p	p	nd	nd	nd	extrafoveal	FF/YD	inside the vascular arcades	nd
49	63	F	RE	1/40	0.00	cataract	nd	nd	p	nd	p	nd	foveal	FF/YD	posterior pole atrophy	nd
			LE	1/40	0.00	cataract	nd	nd	p	nd	p	nd	foveal	FF/YD	posterior pole atrophy	nd
50	63	M	RE	4/10	-3.00	cataract	p	p	p	nd	nd	nd	foveal	nd	inside the vascular arcades	nd
			LE	1/20	-3.00	cataract	p	p	p	nd	nd	nd	foveal	nd	posterior pole atrophy	nd
51	65	F	RE	1/10	-2.00	N	nd	nd	p	nd	p	nd	foveal	UN	posterior pole atrophy	nd
			LE	1/50	0.50	N	nd	nd	p	nd	p	nd	foveal	UN	posterior pole atrophy	nd
52	68	F	RE	1/20	0.00	cataract	p	p	p	nd	nd	nd	foveal	UN	posterior pole atrophy	nd
			LE	5/10	0.00	cataract	p	p	p	nd	nd	nd	foveal	UN	inside the vascular arcades	nd
53	69	M	RE	hand motion	0.00	cataract	nd	nd	p	nd	p	nd	foveal	UN	posterior pole atrophy	nd
			LE	hand motion	0.00	cataract	nd	nd	p	nd	nd	nd	foveal	UN	posterior pole atrophy	nd

AS: angioid streaks; BD: butterfly shaped dystrophy; CL: comet lesion; CO: *coquille d'oeuf*; EqS: splerical equivalent; FF/YD: *fundus flavimaculatus*/yellowish deposits; FP: *fundus pulverulentus*; N: normal; nd: not detectable; p: present; PD: *Pattern dystrophy*; PO: *Peau d'orange*; UN: unclassified; VD: vitelliform dystrophy.
*Patient #45 was also affected by glaucoma
