# Supplementary material for: Phenotypic Features and Genetic Findings in a Cohort of Italian Pseudoxanthoma Elasticum Patients and Update of the Ophthalmologic Evaluation Score
Source: J Clin Med. 2021 Jun 19;10(12):2710. doi: 10.3390/jcm10122710 (PMC8235548; doi:10.3390/jcm10122710)
Supplement: Supplementary file 1 [file jcm-10-02710-s001.zip › Table S4.pdf]

Table S4. Frequency of singular ocular manifestations according to the characteristics of rare sequence variants detected in 53 PXE patients who underwent in-depth ophthalmological investigation. Group M= two missense variants; group NF= two sequence variants causing a reduced protein production or a protein to be likely non-functional; group M+NF= one missense and one "NF" variant.

Ocular manifestation	Group NF (n=25)	Group M+NF (n= 19)	Group M (n=9)
Peau d'orange/ Coquille d'oeuf	21 (84%)	16 (84%)	8 (89%)
Angioid streaks	23 (92%)	17 (89%)	9 (100%)
Comet lesion	21 (84%)	14 (74%)	5 (55%)
Choroidal neovascularization	12 (48%)	11 (58%)	4 (44%)
Pattern dystrophy-like changes	10 (40%)	8 (42%)	4 (44%)
Posterior pole atrophy	7 (28%)	7(37%)	0 (0%)
Optic disc drusen	1 (4%)	0 (0%)	2 (22%)
